# Decoding the Dopamine Signal in Macaque Prefrontal Cortex: A Simulation Study Using the *Cx3Dp* Simulator

**DOI:** 10.1371/journal.pone.0071615

**Published:** 2013-08-12

**Authors:** Isabelle Ayumi Spühler, Andreas Hauri

**Affiliations:** Institute of Neuroinformatics, University of Zurich and ETH Zurich, Zürich, Switzerland; National Research Council of Italy (CNR), Italy

## Abstract

Dopamine transmission in the prefrontal cortex plays an important role in reward based learning, working memory and attention. Dopamine is thought to be released non-synaptically into the extracellular space and to reach distant receptors through diffusion. This simulation study examines how the dopamine signal might be decoded by the recipient neuron. The simulation was based on parameters from the literature and on our own quantified, structural data from macaque prefrontal area 10. The change in extracellular dopamine concentration was estimated at different distances from release sites and related to the affinity of the dopamine receptors. Due to the sparse and random distribution of release sites, a transient heterogeneous pattern of dopamine concentration emerges. Our simulation predicts, however, that at any point in the simulation volume there is sufficient dopamine to bind and activate high-affinity dopamine receptors. We propose that dopamine is broadcast to its distant receptors and any change from the local baseline concentration might be decoded by a transient change in the binding probability of dopamine receptors. Dopamine could thus provide a graduated ‘teaching’ signal to reinforce concurrently active synapses and cell assemblies. In conditions of highly reduced or highly elevated dopamine levels the simulations predict that relative changes in the dopamine signal can no longer be decoded, which might explain why cognitive deficits are observed in patients with Parkinson’s disease, or induced through drugs blocking dopamine reuptake.

## Introduction

The dopamine signal in the prefrontal cortex (PFC) is crucial for working memory as well as for reinforcement learning [1]–[3]. Manipulations of the dopamine level in monkey PFC, by blocking the dopamine receptors or increasing the dopamine levels, have revealed that there is an optimal level for the performance of cognitive tasks involving working memory. Too high or too low levels of dopamine in PFC are detrimental for cognitive performances of monkeys [4]–[6]. Anatomical studies of dopaminergic projections from the midbrain to striatal and cortical areas suggest that dopamine is not released at synapses, but is released into the extracellular space so allowing it to bind to receptors remote from the release sites [7], [8]. Thus a critical aspect of dopamine transmission is the spatiotemporal change of the extracellular dopamine concentration in relation to the firing activity of the midbrain dopaminergic neurons. Differences in the expression level of the dopamine re-uptake transporter and the density of dopaminergic axons innervating subcortical or cortical areas, predict a different spatiotemporal dynamic between those target areas. Models of the dopamine signal in the densely innervated striatum indicate that the timing of dopamine release in relation to glutamatergic synaptic activity can provide the selectivity of dopamine as a reinforcement signal [9], [10]. Consistent with these predictions, a bidirectional interaction of activated NMDA- and dopamine D1 receptor has been observed experimentally. When dopamine acts at the D1 receptors, it increases the NMDA conductance. The activated NMDA receptors in turn increase the number of available D1 receptors on the membrane of spines [11]–[13]. This reciprocal interaction has further been proposed to underlie the modulatory effect of dopamine on synaptic plasticity and learning in neuronal networks [14].

To simulate the dopamine transmission in PFC, we used the parameters taken from published data and our own measurements of immunohistochemically labelled dopaminergic axons of layer 3 in prefrontal area 10 [15]. Prefrontal area 10 (frontopolar cortex) is active during highly complex cognitive tasks [16], [17]. The simulations reveal that even at baseline firing levels there is enough dopamine to bind on receptors anywhere in the neuropil. We provide a working hypothesis of how changes in dopamine concentration relative to baseline level could serve as a relative teaching signal for working memory representation and reward-related learning.

## Results

### Tonic Firing to Steady State Level

**Figure 1:**
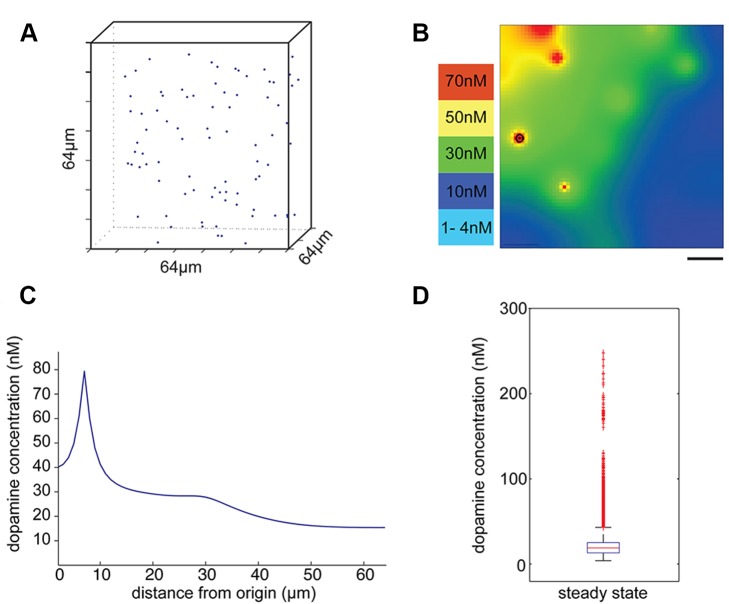
Tonic release of dopamine reaches a steady state level of 26 nM. **A.** Simulation volume with edge length of 64 µm. Dots indicate the location of dopamine release sites in the volume. The density of release sites is given by our previous anatomical measurements. The random distribution has been derived from the nearest neighbour analysis [15] **B.** A section through the simulation volume shows local ‘hot-spots’ of dopamine concentration emerging around release sites during tonic firing. The level of dopamine concentration is indicated by different colors. The release site lying within the same plane of section is indicated with a circle. Scale bar = 10 µm **C.** The plot shows the dopamine concentration along a sample line in the simulation volume at steady state. **D.** The dopamine concentration of every compartment (1 µm^3^) is plotted and shows the distribution and range of dopamine concentration across the entire volume at steady state. All sites receive enough dopamine to bind on D1 receptors in their high affinity state. The box is limited by the 25^th^ and 75^th^ percentiles with the median shown as red line and outliers are plotted as red crosses.

At a tonic background firing of 5.6 Hz and a re-uptake constant of 1.5 s^−1^, the average dopamine concentration reached at steady state was 26 nM (Std: 10.5 nM, Range: 9.5 nM–250 nM). The steady state level lies in the measured range of dopamine concentration in monkey prefrontal cortex [18]. Figure 1A shows the simulation volume with all randomly located release sites. Due to the sparse distribution of dopamine release sites, the concentration varies by a factor of 25. Figure 1B shows a 2-D cross-section through the cubic volume at steady state. The local heterogeneity of dopamine concentration is also shown in Figure 1C, where the dopamine concentrations are plotted along an arbitrary line drawn through the volume. The dopamine concentration at steady state varies between 9.5 nM up to 250 nM (Boxplot Figure 1D), which reveals that at the baseline levels maintained during tonic release, the dopamine receptors in their high affinity state are activated everywhere in the volume.

### Change after Bursts and Depressed Activity

To simulate the change in activity after an unexpected or omitted reward, we examined three different simulation conditions: an increase in firing up to 15 Hz for 150 ms followed by a pause of 0 Hz for 150 ms, an increased firing up to 26 Hz for 150 ms and a depressed activity of 0 Hz for 150 ms. As the number of dopaminergic neurons projecting to PFC with synchronous, phasic firing is not known, we varied the proportion of release sites responding to phasic activation from 10%, 25% and 50%. All the other release sites maintained tonic release. The measured values of all simulation conditions are summarized in Table 1.

**Table 1:** Summary of the simulation results obtained from the different protocols.

	Values (nM)				Abs. Difference (nM)				Rel. Difference (%)				
	Mean	Std	Min	Max	Mean	Std	Min	Max	Mean	Std	Min	Max	
**Tonic**	**26**	10.5	9.5	250									
**15** **Hz**													
10%	**27**	11.5	9.5	441	**0.9**	3	0.01	262	**2.9**	7.4	0.005	147	
25%	**28**	13	9.5	586	**2.2**	4.7	0.01	344	**7.16**	10.3	0.02	149	
50%	**30.5**	16	9.7	586	**4.5**	7.1	0.02	344	**14.4**	15	0.05	155	
**26** **Hz**													
10%	**28**	13.5	9.5	746	**1.9**	6.5	0.01	568	**6.3**	16.2	0.006	320	
25%	**31**	17.6	9.5	988	**5**	10.2	0.01	746	**15.5**	22.4	0.04	322	
50%	**36**	23.4	9.9	989	**9.6**	15.5	0.03	746	**31.2**	32.8	0.1	335	
**0** **Hz**													
10%	**25.6**	10.3	9.5	250	**−0.5**	1.8	−156	0.01	**−1.7**	4.5	−88	0.13	
25%	**24.8**	9.6	9.5	249	**−1.3**	2.8	−204	0.01	**−4.2**	6.2	−89	0.13	
50%	**23.5**	8.6	9.4	246	**−2.6**	4.2	−205	0.009	**−8.5**	9.0	−92	0.07	
**MPTP**													
**Tonic**	**7.5**	5.6	1.0	223									
**15** **Hz**													
10%	**7.7**	6.4	1.0	443	**0.17**	1.5	0.003	263	**0.9**	4.6	0.002	147	
25%	**8.2**	7.4	1.0	454	**0.69**	2.7	0.003	274	**5.4**	12.3	0.002	161	
50%	**8.9**	9.3	1.0	570	**1.4**	4.4	0.003	348	**9.9**	16.8	0.002	161	

The absolute values of the dopamine concentration at steady state (tonic firing) and after phasic firing (t = 150 ms), the absolute difference in dopamine concentration between tonic and phasic firing and the relative difference in dopamine concentration after phasic release are listed as mean with standard deviation (std). The range is presented with the minima and maxima values.

### Dopamine Concentration at Specific Distances to Release Sites

**Figure 2:**
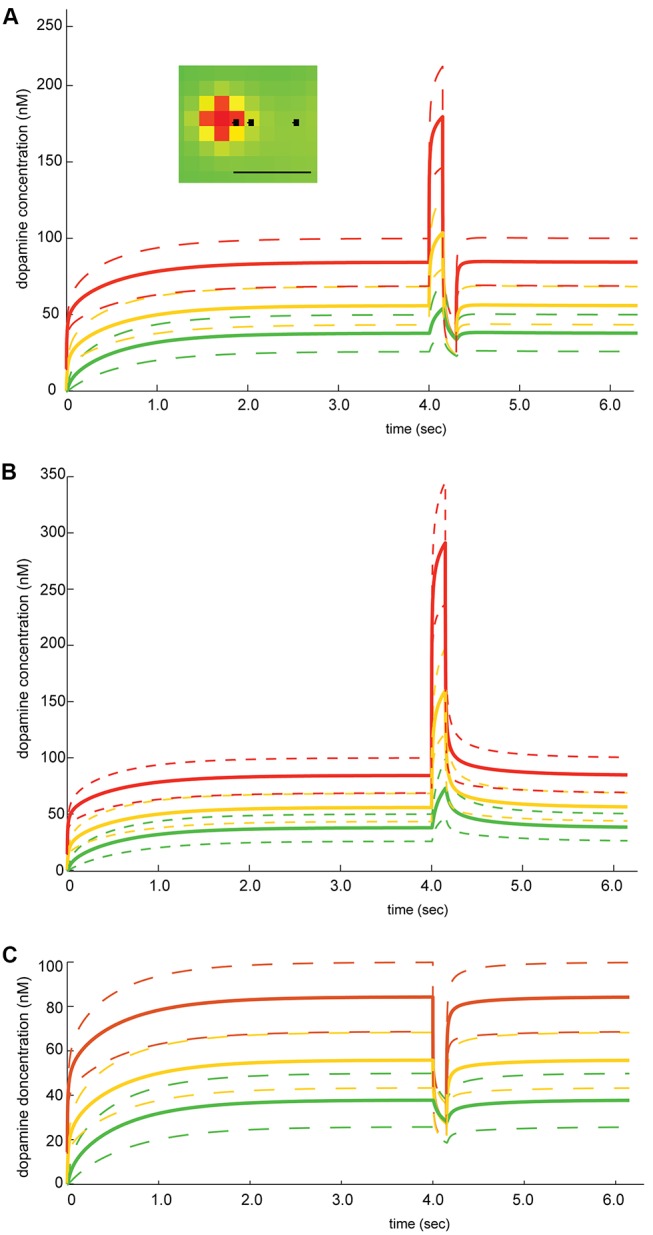
Different simulation protocols of increased firing and depressed activity. Dopamine concentration at different distances from the release sites is plotted against time (red: 1, orange: 2 and green: 5 µm away from release sites, solid lines indicate mean value and dashed lines the standard deviation) Inset illustrates the measurement points with scale bar = 5 µm. In all the different simulation protocols there is tonic release for 4 sec. During this initial period the uptake and release of dopamine reach a steady state level of local dopamine concentration. After 4 sec, 50% of release sites increase or decrease their release for a short time window to simulate phasic firing of the dopaminergic neurons. The firing activity then returns to tonic activity and a local steady state level. The phasic firing is varied in A, B and C. **A.** Phasic firing of 15 Hz followed by depressed activity of 0 Hz, each 150 ms **B.** Phasic firing of 26 Hz for 150 ms. **C.** Depressed activity (0 Hz) for 150 ms. Varying the phasic activity across A, B and C, shows that changes in dopamine concentration depend on the distance to a release site, but that even 5 µm away from a release site a change in the dopamine level is evident.

The change in dopamine concentration over time was measured at three different measurement points: 1, 2 and 5 µm (red, yellow resp. green line) away from every release site with phasic activity. The mean value (solid lines) with its standard deviation (dashed lines) is plotted along time for the respective simulation protocols in Figure 2. The graphs show the amount of dopamine available for the receptors to bind at different distances to a release site. Thus, the simulation predicts that even 5 µm away from release sites with phasic activity, there is a considerable transient change in dopamine concentration after a change in firing frequency.

### Local Heterogeneity

**Figure 3:**
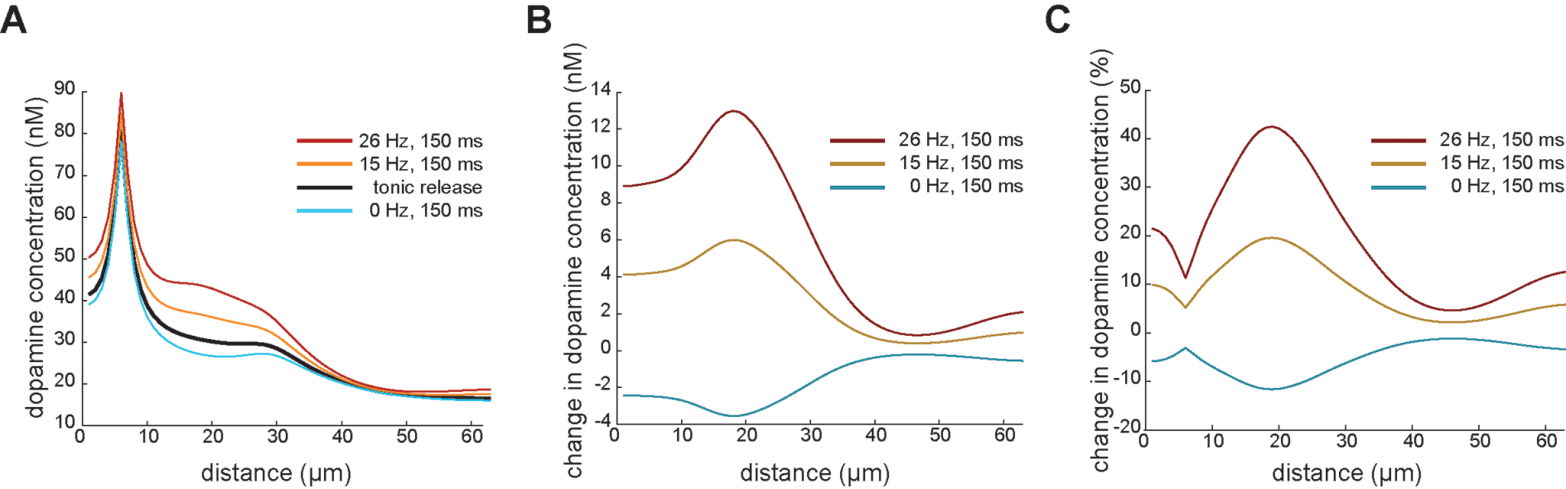
Local change in dopamine concentration from steady state after phasic activity plotted along a sample line through the simulation volume. **A.** Local dopamine concentration is plotted at steady state (black) and after 150 ms of increased (orange: 15 Hz, red: 26 Hz) or depressed (blue, 0 Hz) firing rate along a random line of the simulation volume. **B.** The local difference in absolute dopamine concentration between steady state and that resulting from increased or decreased activity is plotted for the different protocols. **C.** The local difference in the dopamine concentration is plotted as percentage to the baseline level.

**Figure 4:**
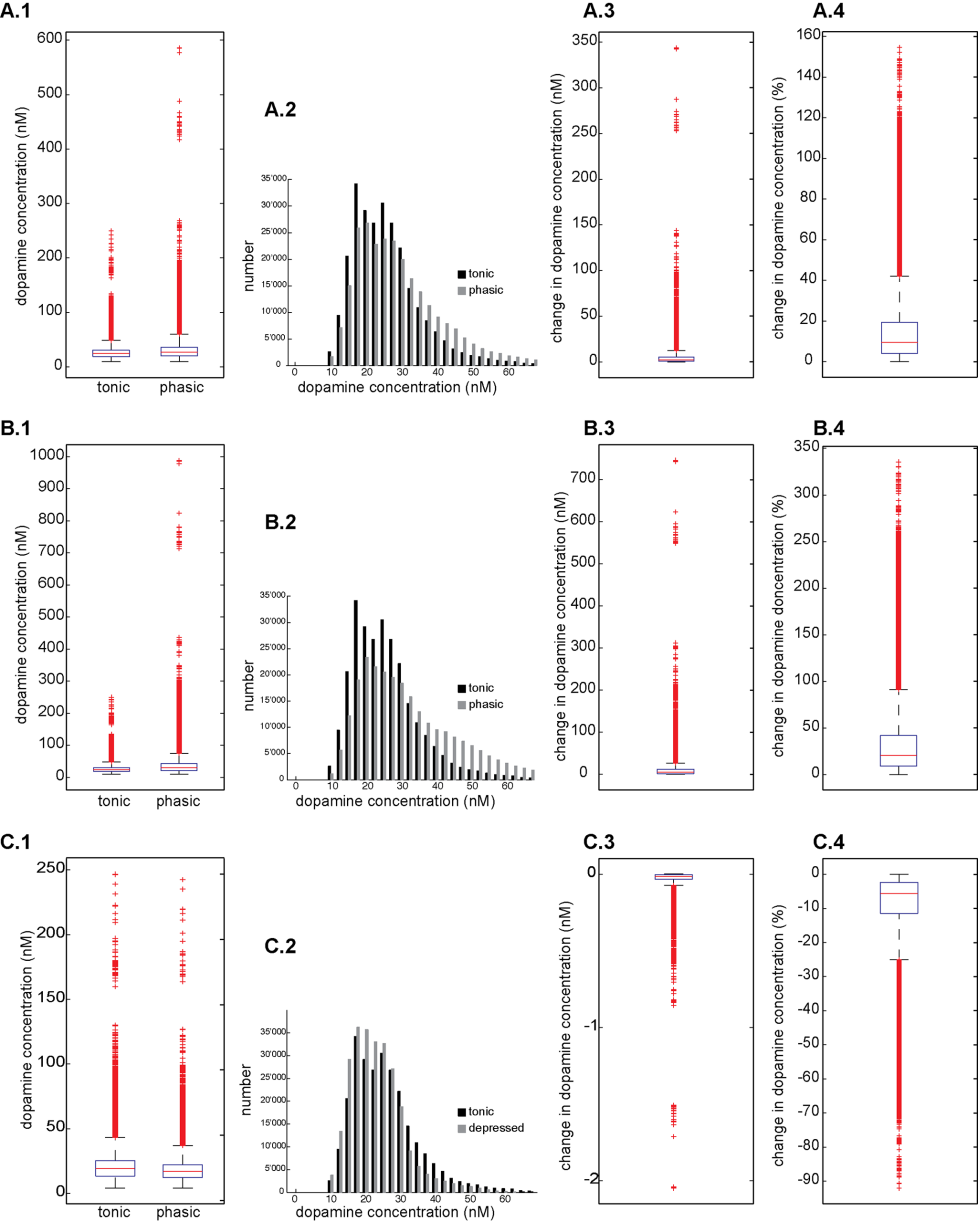
Change in dopamine concentration between steady state and phasic activity within the entire simulation volume. Simulations were performed with 50% of the release sites firing at 15 Hz (A), 26 Hz (B) and 0 Hz (C) during 150 ms. **A–C. 1** The distribution of dopamine concentration during steady state and after 150 ms of phasic activity is plotted (resolution: µm^3^). **A–C. 2** Histogram of the dopamine concentration in the volume of the lower nM range, containing most values. The distribution of local dopamine concentration is plotted during tonic activity at steady state (black) and after phasic activity (grey). **A–C. 3** Change in absolute dopamine concentration in the simulation volume for every µm^3^ after phasic activity. **A–C. 4**. The relative increase of the dopamine concentration after phasic activity is plotted for the different protocols as percentage of steady state.

When we look at the changes in dopamine concentration along a sample line in the simulation volume (Figure 3), we see the local variability in dopamine level as a result of all the release sites with different activity at various distances. The transient local change in dopamine level is variable and not necessarily most pronounced at the sites with the highest absolute dopamine level. For every simulation condition (with 50% synchronised release), the change of dopamine concentration of the entire volume is plotted in Figure 4 (resolution of measurement 1 µm^3^). The relative change in dopamine level varies strongly across the volume. For example, the dopamine concentration increases 14.4% on average over the volume after the phasic activity of 15 Hz (Figure 4, A.4). Very close to a release site with phasic activity the dopamine concentration reaches more than double its baseline level and yet approximately 5% of the volume has a relative increase of less than 1% of its baseline level (see Table 1). In summary, the tonic and phasic release of dopamine results in a heterogeneous pattern with local ‘hot-spots’ of increased dopamine levels, yet all the protocols result in values above the effective concentration required to bind on D1 receptors in their high affinity state everywhere in the neuropil.

### Altered Dopamine Release or Uptake

#### Highly reduced density of dopamine release sites

**Figure 5:**
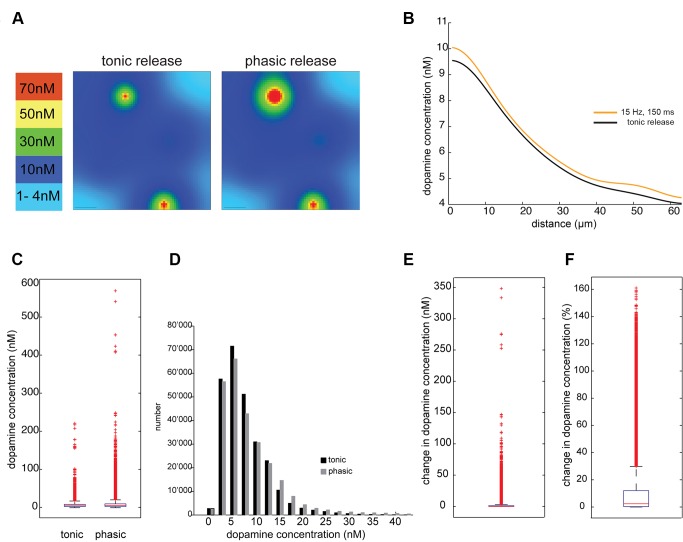
Simulation of Parkinsonian-like condition based on few release sites. **A.** Section through the simulation volume shows the dopamine concentration during tonic release at steady state and after 150 ms of phasic activity (15 Hz) at 50% of release sites in the Parkinsonian-like condition. The dopamine concentration increases close to the release site with phasic activity, however the dopamine level stays mainly below 10 nM. Scale bar = 10 µm **B.** The plot shows the dopamine concentration along a line in the simulation volume at steady state with tonic release and after 150 ms of phasic activity (orange). **C.** Dopamine concentration (resolution: µm^3^) at steady state and after 150 ms phasic activity resulting in increased release. **D.** Histogram shows the distribution of dopamine concentration in the lower nM level at steady state during tonic release and after 150 ms phasic activity. Most values are lower than 10 nM and are thus below high affinity level of dopamine receptors. **E.** The absolute change of the dopamine concentration in the volume (resolution: µm^3^). Mean of the absolute change in dopamine concentration after 150 ms of phasic activity for 50% of release sites is only 1.4 nM (mean value). **F.** Change in dopamine concentration is given as percentage from steady state level (resolution: µm^3^).

**Figure 6:**
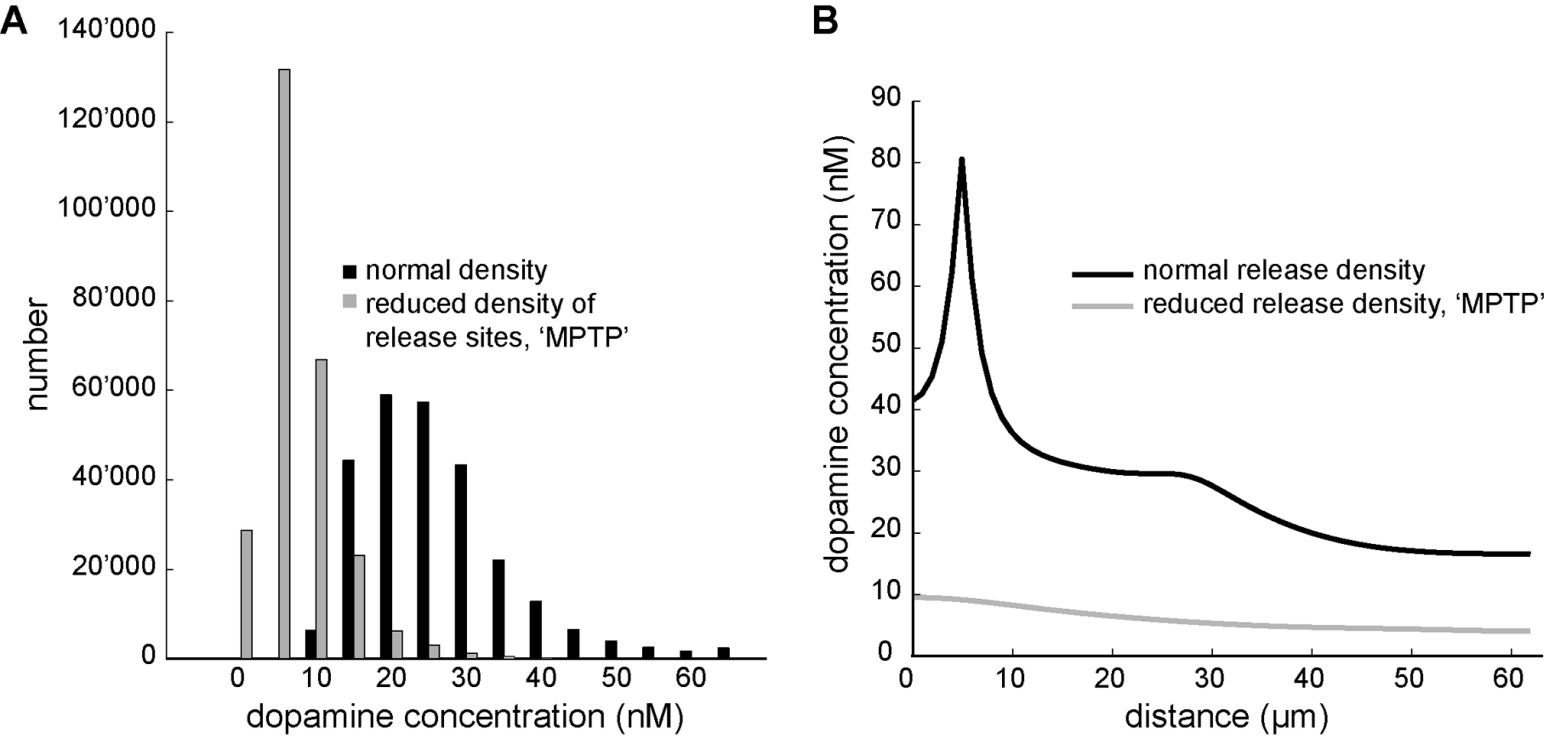
Comparison of local dopamine concentrations at steady state between the two conditions with normal and highly reduced number of release sites. Simulation shows the steady state difference of local dopamine concentration for a normal density of dopamine release sites and with highly reduced number of release sites. A) Histogram shows the distribution of local dopamine concentration in the two simulation conditions. Most sites within the volume in the MPTP condition show a dopamine concentration lower than 10 nM while in the normal condition most sites are above 10 nM. B) A random sample line within the simulation volume shows the local dopamine concentration and its local variation. The black sample line is taken in the simulation volume with normal number of release sites and the grey line is sampled from the simulation volume with highly reduced numbers of release sites.

In primate models of Parkinson’s disease, dopamine depletion is typically induced by treatment with the selective neurotoxin methyl-4-phenyl-1,2,3,6-tetrahydropyridine (MPTP). It has been reported that the dopamine level and innervation density in prefrontal areas of MPTP treated monkeys is reduced by 40–74% [19], [20]. Our unpublished observations of reduced dopamine innervation in area 10 of MPTP treated monkeys indicate MPTP effects on the density of release sites can be even more severe. We assumed a depletion of 70% of release sites in our model, which resulted in a steady state level of dopamine concentration of 8 nM assuming innervation density decreased to 0.61 *10^−4^ release site/µm^3^. Simulation condition with phasic firing of 15 Hz for 150 ms and depressed activity of 0 Hz for 150 ms has been simulated. Figure 5 summarizes the dopamine concentration in the Parkinsonian-like condition. The dopamine level is very low and during tonic firing 42% of the volume receives less than 5 nM and only 22% of the volume reaches more than 10 nM (Figure 5 A–D). If the effective concentration for the D1 dopamine receptors in the PFC is 10 nM, this would mean that most of the volume does not receive sufficient dopamine to activate their high affinity receptors (Figure 5D). The mean of absolute change in dopamine concentration after phasic activity is only 1.4 nM (Figure 5E and Table 1) and though the relative change is on average almost 10% (Figure 5F and Table 1), as the steady state level is very low, the dopamine concentration in the entire volume stays low even after phasic release (Figure 5D). The difference of extracellular dopamine concentration between the two conditions with normal or highly reduced number of release sites is shown in Figure 6. The histogram in Figure 6A shows the distribution of the local concentration (without outliner values) at steady state, during tonic release of the two conditions. Sampling from a random line within the volume also shows the decrease in extracellular dopamine concentration in comparison to the normal condition (Figure 6B).

#### Drug-induced block of dopamine re-uptake

**Figure 7:**
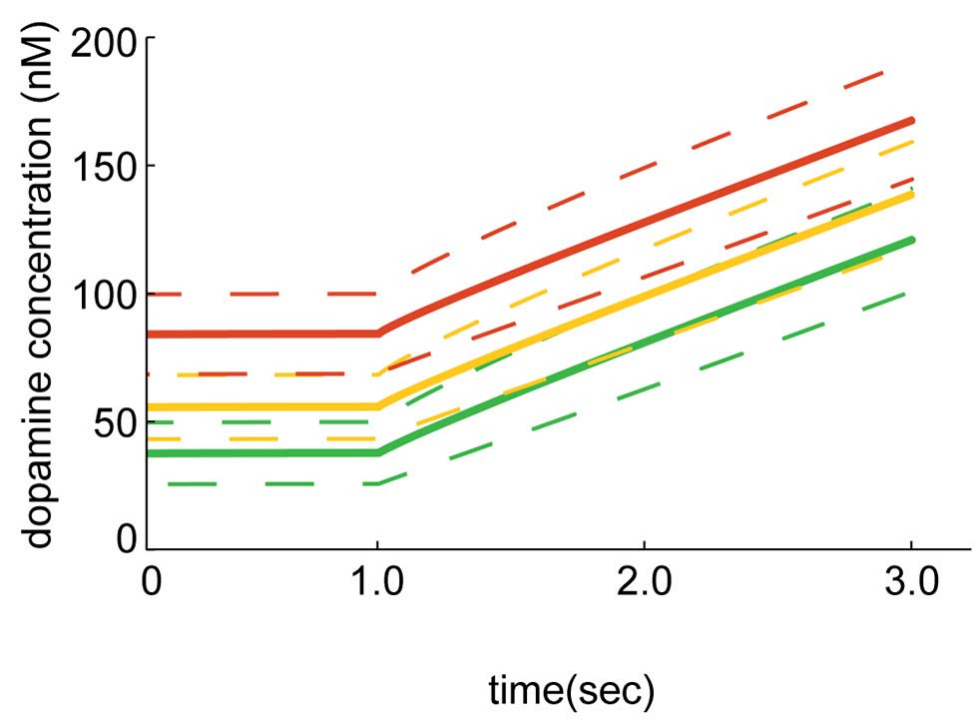
Simulation performed with blocked dopamine re-uptake. The mean dopamine concentration at a distance of 1 µm (red), 2 µm (yellow) and 5 µm (green) from a release site is plotted against time. The dashed lines indicate the standard deviation. The local dopamine concentration reaches steady state due to tonic release and linear re-uptake of dopamine. At time t = 1.0 sec all the re-uptake is blocked while the release sites keep their tonic release activity. The dopamine concentration increases on average 39 nM per second and would reach µM level after 20–25 seconds.

The dopamine re-uptake transporter is blocked by drugs such as Amphetamine, Methylphenidate (Ritalin) or Benzoyl-methyl-ecgonine (Cocaine) [21] and we tested in our simulation volume a condition with a completely blocked re-uptake of dopamine. Our simulation does not consider the biochemical and pharmaceutical dynamics of the transporters and drug and we are aware that drugs do not lead to a complete block of the transporters. However, the simulation can be refined by adjusting the depressed uptake dynamics. If the re-uptake is blocked completely and the tonic release activity is kept constant the dopamine concentration increases on average 39 nM per second and would reach µM levels after 20–25 sec (Figure 7). The change in local dopamine concentration is plotted at the three measurement points: 1, 2 and 5 µm (red, yellow resp. green line) away from every release site. At micromolar levels the dopamine receptors in their resting, i.e. low affinity, state would bind to dopamine. The dopamine receptors in their resting state are not bound to the G-protein and are thus ineffective in evoking intracellular signalling. However, at micromolar levels the dopamine receptors would bind to dopamine in both conformational states.

## Discussion

By simulating dopamine diffusion in prefrontal area 10 we could show that non-synaptic release of dopamine leads to local heterogeneity of dopamine concentration due to the low density of the release sites. During the phasic release of dopamine the ‘hot-spots’ enlarge and the overall dopamine level increases. Our results address important questions about how dopamine neurons encode their signal, how this signal is decoded by the recipient neurons in the PFC and also, by what means cell assemblies and individual synapses are modulated?

### Sparse Innervation and Local ‘Hot-spots’ of Increased Dopamine Levels

In contrast to the striatum, where any given structure of the neuropil lies within a radius of 1 µm [22], of a dopaminergic bouton the nearest neighbour distances between potential dopamine release sites in area 10 of macaque PFC is on average 9 µm [15]. This sparse innervation in primate neocortex raises the question of whether there is a restricted sphere of neuropil within which the dopamine concentration is effective and if so, whether the composition of the neuropil within this sphere is special or different from the neuropil distant from a release site. The effective radius for dopamine to bind on the high affinity receptor after a single release event in the striatum has been predicted to be around 7–8 µm [23] or even more than 20 µm [24]. Based on the tonic and phasic activity of several release sites we examined the local variation of dopamine concentration within the extracellular space. In line with the prediction of the effective radius after a single release event, our simulations showed that the tonic activity of all the release sites generates a dopamine concentration high enough to activate all the dopamine receptors in their high affinity state anywhere in the neuropil. Even though there are ‘hot-spots’ of high dopamine concentration centred on release sites, the dopamine signal is broadcast with sufficient concentration to all sites of the neuropil. There are no regions where the dopamine concentration is too low to be effective.

### Local Variability within the Nanomolar Range

Although there is enough dopamine to activate receptors in their high-affinity state throughout the neuropil, the sparse innervation nevertheless results in dopamine concentrations that range over tens to several hundreds of nanomolar. ‘Hot-spots’ of increased dopamine concentration occur, but the simulations demonstrate that, unlike the dopamine level in the simulation of the striatum [10], micromolar levels are not reached in the prefrontal cortex. Micromolar concentrations of dopamine would have the consequence that the receptors would be bound to dopamine even in their resting conformation (low affinity state). The local variation of dopamine concentration in our cortical simulation stays below micromolar levels, which implies that the receptors in their low affinity state never bind dopamine, but in their high affinity state do bind with a variable probability according to a decrease or increase in local dopamine concentration.

### Working Hypothesis of Decoding the Dopamine Signal

From the predictions of our simulation, we propose it is not the absolute value of dopamine, but the relative change of dopamine level that is detected against the baseline level of dopamine maintained by the tonic firing. Given that during tonic firing dopamine reaches a sufficient concentration to be bound by receptors in their high-affinity state, an increase or decrease in the concentration of dopamine principally affects the binding probability of the dopamine receptors and eventually the number of activated dopamine receptors. The crucial signal for the recipient site is hence not the absolute level, but whether there is more or less dopamine available relative to the local baseline level. Physiological recording of dopaminergic neurons indicate that they have three firing patterns: tonic firing as background activity, bursts of firing after the occurrence of an unexpected reward, and depressed activity of the neurons after an omission of an expected reward [25]. The prediction error associated with the firing pattern of dopaminergic neurons would be reflected in the change in dopamine concentration from its baseline level. A phasic increase or decrease of extracellular dopamine due to phasic firing or absence of release would change the binding probability of the available receptors, and thus the number of activated dopamine receptors. In this way the dopamine signal can be encoded and decoded in a graduated manner in relation to its baseline signal.

### Dynamic Balance between the Affinity States

The two-state model of G-protein coupled receptors proposes that there is a dynamic balance between the high and low affinity state [26]. The equilibrium constant between the conformational states can alter the probability of a receptor being in the high or low affinity state. Richfield and colleagues [27] described the rodent striatum as having 80% of the D1R in their low affinity state and only 20% in their high affinity state. We propose that the relative proportion of dopamine receptors in their high or low affinity states is locally adjusted in order to account for the local heterogeneity of dopamine concentration and thereby to ensure an optimal detection of any change relative to the steady state level at any site in the neuropil. There are several other ways to regulate the total number of receptors: more receptors recruited on the membrane, less inactivation of receptors, or restricted lateral diffusion of receptors within the membrane. This suggests a high degree of flexibility on the part of the recipient synapse to adapt to the prevailing dopamine concentration by regulating the number of dopamine receptors in the high-affinity state.

### Condition of Altered Dopamine Signalling

Our simulation of the Parkinsonian-like condition predicts that when only 30% of release sites are active, a detectable and effective dopamine concentration is provided only for receptors close to the release sites and not for the entire volume. On the other hand, when drugs block re-uptake the resultant increase in dopamine level to micromolar concentrations means that dopamine receptors can be occupied in their low affinity state. The dopamine receptors would then be bound to their ligand in both affinity states and no transient change in dopamine concentration would be detected as a change in binding probability of the dopamine receptors. For both very low numbers of release sites, or blocked re-uptake, a graduated dopamine signal would be no longer possible. Our simulation thus explains the inverted U-shaped relationship of dose-dependency for dopamine efficacy [5]. This relationship has direct relevance for the cognitive impairments, especially in working memory, as observed in Parkinson’s disease and in drugs that block dopamine re-uptake.

### Combination of Activity State and Change in Dopamine Level Ensures Specific Action

A reciprocal interaction has been described between the NMDA and D1 receptors [11], [13], [28]. NMDA receptor activation increases the recruitment of D1 receptors and thus the sensitivity to dopamine. On the other hand D1 receptor activation enhances the excitatory postsynaptic current mediated by the NMDA receptors. There is thus a bidirectional interaction between the two receptors and reciprocal signal enhancement. This synergy between the dopamine D1 receptor and NMDA receptor is hypothesized to be an important mechanism for synaptic plasticity [14], [29]. Put in relation to the results of our simulation, this observation suggests the mechanism whereby the broadcast dopamine signal can nevertheless act specifically. The simulation shows that dopamine can affect any synapse in the neuropil, however the reciprocal interaction between activated NMDA and D1 receptors implies an enhanced action of dopamine only on glutamatergic synapses that are coincidently active during increased dopamine release. Bursts of firing are associated with an unexpected reward and the increase in dopamine would thus enhance currently active cell assemblies. The combination of the activity state of the recipient synapse and the change in dopamine concentration could give rise to the specific action of dopamine for synaptic plasticity. With this view the role of dopamine as a teaching signal is optimally provided by a non-synaptic signal, broadcast to all synapses in the neuropil and yet to act specifically due to dopamine receptors and their interactions.

### The Role of Dopamine Receptors in Signal Transmission

In the current study we were primarily interested in examining the spatiotemporal dynamic of the dopamine signal in the prefrontal cortex where the dopaminergic innervation is very sparse. However, these investigations could be extended within the simulation framework of *Cx3Dp* to include detailed neuronal structures with synaptic connectivity into the simulation volume, which would allow an exploration of the dynamics as well as plasticity of cell assemblies in relation to a change in dopamine concentration associated with a ‘teaching’ signal. Our simulations could also be extended to refining theoretical models of dopamine signal on working memory representation. We propose a central role of the dopamine receptors in the transmission of dopamine: dopamine is provided to all synapses in the volume and act on all structures in the neuropil that express the receptors necessary to receive the signal. Active synapses in a given cell assembly could be enhanced by means of the bidirectional interaction of dopamine-activated D1 receptors and NMDA receptors. Our study reveals the importance of a more detailed understanding of the dopamine receptors, their affinity states, and their interaction with other receptors and neurotransmitter systems.

## Methods

### Cx3Dp, the Simulation Tool

The simulation of the diffusion was performed using *Cx3Dp*, a simulation framework that provides a three dimensional space to study biological processes with physical rules (http://www.ini.uzh.ch/~amw/seco/cx3d/). It was originally developed to model cortical growth and development [30] but was well suited to the present study. Diffusion of chemical factors is a basic function integrated into *Cx3Dp* and is applied to different biological growth processes. For the simulation of dopamine diffusion in cortex it was necessary only to adjust the diffusion process with specific parameters for uptake and release of dopamine.

### The Simulation Volume

The diffusion of dopamine is simulated in a cubic space representing an idealized block of cortical area 10, layer 3 with a volume of 0.0026 mm^3^ (64 µm on each side). In *Cx3Dp*, the space is divided into discrete compartments (1 µm^3^). Diffusion has been implemented with Fick’s Law as 1^st^ order Euler step function with finite element methods across the compartments. The detailed composition of the neuropil was approximated by assuming the extracellular fraction of the volume was 23% and had a tortuosity of 1.6 [31].

### The Release Sites

The density of release sites was measured on immunohistochemically stained tissue of monkey area 10 [15]. As these axons formed very rarely synapses, putative release sites were defined as regions along the axon where vesicles clustered. These clusters were located principally in boutons. A density of 0.0018 vesicle-filled profiles/µm^2^ was determined from ultramicrographs in layer 3. Considering an average bouton diameter of 0.9 µm, a 3-D density of 0.0002/µm^3^ was obtained. A volume of 0.0026 mm^3^ contained 52 randomly located release sites with an average nearest neighbour distance of 9 µm. As the arbor of a single dopaminergic axon projecting to the cortex has not been characterised yet and the average interbouton distance or bouton density on a single dopamine axon is not known, we assumed release occurred independently at all sites.

Dopaminergic cells have a tonic background firing rate of 5.3±1.5 Hz [32]. Phasic firing of 15–26 Hz and depressed firing of 0 Hz (pause) were observed after an unexpected reward or the omission of a reward [3], [33]. This was captured in the simulation by phasic activity of 10, 25 or 50% of release sites in synchrony (Table 2). The remaining release sites remained at tonic firing level. No measurements about the release probability of dopamine in primate cortical areas was available in the literature at the time we performed our simulation, so we assumed a release probability of 0.5. Very recently however the release probability of dopaminergic neurons of mouse VTA has been reported to be on average 0.25, with a range of 0±0.02 to 0.73±0.01 [34]. Our assumed value was thus within their measured range, though higher than their average measure.

**Table 2:** List of different simulation conditions applied.

**A**	Phasic 15 Hz, 150 ms; 0 Hz, 150 ms	0.0002 release sites/µm^3^, reuptake rate 1.5 s^−1^
**B**	Phasic 26 Hz, 150 ms	0.0002 release sites/µm^3^, reuptake rate 1.5 s^−1^
**C**	Pause 0 Hz, 150 ms	0.0002 release sites/µm^3^, reuptake rate 1.5 s^−1^
**D**	Phasic 15 Hz, 150 ms; Pause 0 Hz, 150****ms	0.000061release sites/µm^3^, reuptake rate 1.5 s^−1^
**E**	Only tonic	0.0002 release sites/µm^3^, reuptake blocked

All the simulations had a tonic firing of 5.6 Hz and release probability of 0.5 during the first 4 sec. For the following phasic activity, different firing rates were applied. For every condition A–D, 10, 25 or 50% of the release sites showed phasic or depressed activity.

### The Uptake Sites

Immunohistochemical studies of dopaminergic receptors and transporters indicated that they are expressed distant to putative release sites or synapses [7], [35]–[37]. The low expression level of dopamine transporters and comparisons of the dopamine signal between striatum and cortex led to the hypothesis that the uptake rate in the PFC was ten times slower than in the striatum [36]. The striatal uptake rate has been described to be 20 s^−1^ [23] and we set the cortical uptake rate to 1.5 s^−1^. For the purpose of the simulation the dopamine receptors and transporters were uniformly distributed in the space and thus the uptake was modelled to occur uniformly through the volume. We were interested in the activation of dopamine receptors D1 and D2 in relation to the dopamine concentration during tonic background activity and after the transient phasic release. D1 and D2 of the striatum are thought to be in high or low affinity states [27]. The affinity states of the G-protein coupled dopamine receptors change with their conformational states and have values of low nM in the high affinity state (effective conformation) and few µM for the low affinity state (resting conformation) [38]. There are no measurements of the affinity state and their dynamic balance between the effective and resting state for the dopamine receptors of the primate prefrontal cortex, so we used the same values as assumed for the striatum in our cortical model.

All numeric values of the applied parameters and the corresponding references are given in the table below (Table 3).

**Table 3:** List of parameters applied in the simulation of dopamine diffusion and their references.

Description	Value	Reference
Density of potential release sites	2/10000 µm^3^	[15]
Volume of simulation	64×64×64 µm^3^	
α, extracellular space %	0.23	[31]
Vesicle volume	6.5*10^−20 ^l	[39], [23]
Cv, [DA]_vesicle_	0.25 M	[23]
Quantity in vesicle	1.625*10^−20^ ****moles	[40]
Tonic firing rate	5.6 Hz	[3], [32], [33]
Phasic firing rate	15 Hz	
Uptake rate constant rat striatum	20s^−1^	[41]
Uptake rate constant mk PFC, *k*	1.5s^−1^	About ten times less than striatum
Release probability	50%	[34]
[DA] during tonic release	25–30 nM	[18]
Dopamine diffusion constant	763 µm^2^ s^−1^	[41]
Tortuosity	1.54	[31], [41]
Effective dopamine diffusion constant	322 µm^2^ s^−1^	[23], [42]
D1 receptor affinity	∼10 nM high affinity 1–2 µM low affinity (70%in low affinity)	[23], [27], [43]
D2 receptor affinity	∼10 nM high affinity 1–5 µM low affinity (20%in low affinity)	[23], [27], [43]
